# The German version of the Perceived Stress Scale – psychometric characteristics in a representative German community sample

**DOI:** 10.1186/s12888-016-0875-9

**Published:** 2016-05-23

**Authors:** Eva M. Klein, Elmar Brähler, Michael Dreier, Leonard Reinecke, Kai W. Müller, Gabriele Schmutzer, Klaus Wölfling, Manfred E. Beutel

**Affiliations:** Department of Psychosomatic Medicine and Psychotherapy, University Medical Center of the Johannes Gutenberg University Mainz, Untere Zahlbacher Str. 8, 55131 Mainz, Germany; Department of Communication, University of Mainz, Jakob-Welder-Weg 12, 55099 Mainz, Germany; Department of Medical Psychology and Medical Sociology, University of Leipzig, Philipp-Rosenthal-Straße 55, 04103 Leipzig, Germany

**Keywords:** PSS, Stress, Psychometric properties

## Abstract

**Background:**

The Perceived Stress Scale Cohen (J Health Soc Behav 24:385-96, 1983) is a widely and well-established self-report scale measuring perceived stress. However, the German version of the PSS-10 has not yet been validated. Thus, the purposes of this representative study were to psychometrically evaluate the PSS-10, and to provide norm values for the German population.

**Methods:**

The PSS-10 and standardized scales of depression, anxiety, fatigue, procrastination and life satisfaction were administered to a representative, randomly selected German community sample consisting of 1315 females and 1148 male participants in the age range from 14 to 90 years.

**Results:**

The results demonstrated a good internal consistency and construct validity. Perceived stress was consistently associated with depression, anxiety, fatigue, procrastination and reduced life satisfaction. Confirmatory factor analysis revealed a bi-dimensional structure with two related latent factors. Regarding demographic variables, women reported a higher level of stress than men. Perceived stress decreased with higher education, income and employment status. Older and married participants felt less stressed than younger and unmarried participants.

**Conclusion:**

The PSS-10 is a reliable, valid and economic instrument for assessing perceived stress. As psychological stress is associated with an increased risk of diseases, identifying subpopulations with higher levels of stress is essential. Due to the dependency of the perceived stress level on demographic variables, particularly age and sex, differentiated norm values are needed, which are provided in this paper.

## Background

Stress and its consequences on health has been a major research topic over the last decades. Psychological Stress is considered as a crucial factor in the onset, course and exacerbation of various diseases, e.g. depression, cardiovascular diseases, immune-related disorders, and it has been related to higher overall mortality [1–5]. Overall, perceived stress is linked to reduced life satisfaction [6, 7]. While there is agreement on the substantial impact of stress on health, the conceptualization and assessment of stress, however, have not been consistent. In line with different definitions, the concept of stress has been assessed from environmental, biological, and psychological perspectives [8]. Besides the environmental and biological approach, the psychological approach has focused on the person’s appraisal of the significance of the stressor (primary appraisal) and the individual coping abilities (secondary appraisal) within a person-environment transaction [9].

The Perceived Stress Scale (PSS) developed by Cohen, Kamarck and Mermelstein [10] is a well-established self-report measure based on the psychological conceptualization of stress. The scale assesses “the degree to which situations in one’s life are appraised as stressful” (p. 387; [10]). It measures the degree to which life has been experienced as unpredictable, uncontrollable and overloaded in the past month. Cohen and Williams [11] defined perceived stress as a unidimensional construct. As the questions are general in nature, the scale is considered broadly applicable for any population subgroup [12]. The original scale consists of 14 items (PSS-14), however, four items were dropped because of low factor loadings revealed by a principal components analysis. The shortened 10-item scale (PSS-10) showed slightly improved reliability (Cronbach alpha = .78 vs. Cronbach alpha = .75) and equivalent validity [11] and has therefore been recommended for epidemiological and clinical research [11, 13]. For settings with a limited time frame, e.g. telephone interviews, a brief 4-item version (PSS-4) has been developed [11]. However, a recent study exploring the structure of the PSS-4 in a community sample showed insufficient internal validity of the brief four-item version [14].

The PSS has been applied in diverse samples and in numerous studies investigating the association of perceived stress, for example, with human reproduction [15], coping profiles and health-related behavior [16], and cortisol level [17]. In a recent review evaluating the psychometric properties of the PSS across studies from different cultures and countries [13], Cronbach’s α for the 10-item version ranged from .78 to .91. Furthermore, the PSS demonstrated satisfactory test-retest reliability. Concerning construct validity, the PSS correlated moderately to strongly with depression and anxiety. In contrast to the theoretical one-dimensional conceptualization of perceived stress, findings of studies conducting factor analysis suggested and subsequently confirmed a two-factor structure for the PSS-10 across cultures. Regarding demographic characteristics, PSS scores were significantly lower in younger, married, and employed participants with higher income and fewer children. Interestingly, religion was not considered in the studies reviewed although religious coping has been a research topic in the investigation of psychological adjustment to stress [18, 19]. Although Lee [13] claimed that results for sex were inconsistent, the finding of higher perceived stress among women compared to men has been repeatedly replicated [20–22]. Women tend marginally to report more stressful life events [23] and suffer slightly more often from burn-out [24]. In a national US survey using the PSS-10 Cohen and Janicki-Deverts [25] found that women reported more perceived stress than men and stress increased with lower age, education and income. The inconsistent findings on age in this survey compared to the reviewed studies may have been due to different age compositions in the different samples. In regard to employment status, the level of stress was highest among unemployed participants, and lowest among retired persons. In contrast to this study, the majority of the studies reviewed by Lee [13] assessed college students or working populations. Therefore, the author stressed the importance of validating the PSS in representative samples and in various cultures in future studies.

Although the Perceived Stress Scale has become a well-established, world-wide used tool for assessing perceived stress since its introduction in 1983, the PSS-10 has not yet been validated in the German population. Therefore, the purposes of the present paper were (1) to assess the psychometric properties of the PSS-10 (reliability and validity, confirmatory factor analysis), (2) to compare demographic subgroups concerning general perceived stress, and (3) to provide norm values of a representative sample of the German population across the full age range from 14 to 95 years.

In line with previous research, we expected that perceived stress was associated with heightened distress (depression, anxiety, fatigue) and reduced quality of life. The association to procrastination, putting off intended action, as a personal trait consistently linked to stress [26], was also explored in the current study. We assumed that the scale was based on two latent factors. Additionally, PSS scores were hypothesized to be higher in younger age, females, unemployed and single participants.

## Methods

### Participants

The present study was based on a representative survey of the German population. Data were collected by USUMA (Unabhängiger Service für Umfragen, Methoden und Analysen; Berlin) between February and April, 2014. The sample consisted of a total of 2,527 participants (1,350 women; 1,177 men) between the ages of 14 and 95 years who were recruited at 258 sample points, representing East and West Germany; the majority (79.9 %) lived in the Western states of Germany. Participants, who gave informed consent, were interrogated by face-to-face-interviews by trained interviewers in their homes and independently filled out additional questionnaires in the presence of the interviewer. No incentives were offered for study participation. The survey followed ADM (Arbeitskreis Deutscher Markt- und Sozialforschungsinstitute e.V.) sampling guidelines for generating a representative sample of the German population [27]. The sampling procedure comprised three steps: First the areas were regionally stratified (1st step) for identifying sampling points, where private households were selected (2nd step). In the 3rd step the individual within the selected household was determined. By applying this random-route procedure the region, the households and target persons living in the households were randomly selected. After contacting the selected participants in their home, 55.1 % of the initial sample (4,607 households) were interviewed. The resulting quota matched other representative population samples. Subjects who did not complete all ten items of the PSS were excluded from the analysis (*n* = 64). A detailed sample description is given in Table 1 according to sex.

**Table 1:** Sample description

		Total (*N* = 2463)	Women (*n* = 1315)	Men (*n* = 1148)
		%	%	%
Age	mean	49.4	49.9	48.9
	SD	17.7	17.7	17.7
	range	14-95	14-95	14-89
Age groups	14-19	4.5	3.3	5.8
	20-29	12.1	12.5	11.6
	30-39	13.7	14.1	13.2
	40-49	18.6	18.6	18.6
	50-59	19.7	19.9	19.5
	60-69	16.7	15.6	18.0
	70+	14.7	16.0	13.2
Marital status	unmarried	27.2	23.1	31.9
	married	48.2	46.1	50.4
	divorced	13.9	14.8	12.8
	widowed	10.7	15.9	4.8
Children	yes	24.8	27.5	22.0
Migration	yes	3.7	3.7	3.6
Religion	Christian	67.9	70.9	64.5
	Muslim	2.4	1.9	3.1
	other	1.7	2.2	1.0
	no religion	27.9	24.9	31.4
Education	<10th grade	35.2	34.1	36.4
	completed 10th grade	36.9	40.3	32.9
	high school	12.0	9.0	10.1
	college/university	10.0	8.8	11.3
	without graduation	3.2	3.3	3.0
	other	2.8	4.5	6.3
Employment	Employed	54.1	52.1	56.5
	Student/training	6.5	5.5	7.8
	Unemployed	6.1	5.8	6.3
	Retired	28.3	28.4	28.3
	Household	4.0	6.9	0.8
	other	10	1.3	0.3
Income^a^	≤750	16.1	23.7	7.9
	751-1250	30.3	36.6	23.3
	1251-2000	35.1	31.1	39.5
	2001-3500	16.0	7.6	25.2
	>3500	2.6	1.2	4.1

^a^ €/month per person

### Ethics statement

The study, including the consent procedure, was approved by the institutional ethics review board of the University of Leipzig (Az 063-14-10032014). Furthermore, the study adhered to ICH-GCP-guidelines as well as to the guidelines of the ICC/ESOMAR International Code of Marketing and Social Research Practice. All participants were informed of the study procedures, data collection and anonymization of all personal data. Moreover, a detailed data privacy statement was delivered by the study assistant. According to German law, all participants provided verbal informed consent, which was noted by the trained interviewer before starting with the survey.

### Measures

Demographic data included age (≥14 years), sex, marital and employment status, religion, migrant background, education and total income of household.

The German version of the PSS-10 (PSS-10; [10]) was used to measure the degree to which life in the past month has been experienced as unpredictable, uncontrollable and overwhelming (e.g. “In the last month, how often have you felt nervous and "stressed"?) on a 5-point response scale (0 = “never”, 1=”almost never”, 2=”sometimes”, 3=”fairly often”, 4=”very often”). The scale was forward translated from English to German and subsequently back translated by two interdependent bilingual speakers. After reversing the scores on the four positively stated items (Items 4, 5, 7, and 8), a PSS-10 total score was obtained by summing up all 10 items. Higher scores indicated a higher level of perceived stress. As the PSS is not a diagnostic instrument, there are no cut-off scores.

In addition to the PSS-10, socio-demographic questions and additional psychological variables were measured by validated and standardized self-report inventories. These included screening questionnaires for depression and generalized anxiety (PHQ-4), the short form of the General Procrastination Scale (GPS-K), the Copenhagen Burnout Inventory (CBI), and the Life Satisfaction Questionnaire (FLZ-M) during the interview.

The PHQ-4 [28] consists of two items reliably assessing the core symptoms of depressed mood and loss of interest plus two screening items of the short form of the GAD-7 (Generalized Anxiety Disorder [GAD]-7 Scale) : “Feeling nervous, anxious or on edge” and “not being able to stop or control worrying”. The frequency of occurrence in the past two weeks was rated from 0 =”not at all”, 1 =”several days”, 2 =”over half the days”, and 3 =”nearly every day”. Answers of the first two items were added into a total score (0 to 6); a score ≥ 3 has a good sensitivity (87 %) and specificity (78 %) for major depression. Cronbach alpha in the present study was = .83. A sum score ≥ 3 (range 0–6) of the other two items indicates generalized anxiety with good sensitivity (86 %) and specificity (83 %), performing well as a screening tool for all anxiety disorders [29]. The internal consistency in the current study was Cronbach alpha = .77.

Procrastination was assessed by the 9-item short form of the General Procrastination Scale (GPS-K; [30]). Participants rated how characteristic they considered each behaviour (e.g. “I delay the completion of certain things”) on a 4-point scale (1=”very uncharacteristic” to 4 = “very characteristic”). The scale showed good reliability and validity in a representative German community sample [30]. The internal consistency was Cronbach alpha = .92.

The Copenhagen Personal Burnout Inventory (CBI; [31]) is part of the Copenhagen Psychosocial Questionnaire assessing physical and mental exhaustion, independently from work. It assessed the frequency of six items („How often do you feel …“): “tired, physically, emotionally exhausted, unable to go on, weak and prone to illness.” The items were rated on a 5-point scale 1 =”never/almost never”, 2 = “rarely”, 3 = “occasionally”, 4 = “often” to 5 = “always” (COPSOQ; [32]). The scale was reliable (Cronbach alpha in the present study = .91).

The Questionnaire on Life Satisfaction FLZ^M^ [33] is a multi-dimensional self-report measure of individual life satisfaction covering eight relevant areas of life (friends, leisure time activities/hobbies, general health, income, work/career school, housing/living conditions, family life and partnership/sexuality). Additionally, a sum score of all dimensions was used as an index of global life satisfaction. Respondents rated the present satisfaction with these dimensions on a scale from 1 = “dissatisfied” to 5 = “very satisfied”. As the scale bases conceptually on different domains, the life satisfaction sum-scores indicated only sufficient internal consistency (Cronbach alpha = .70).

### Statistical analysis

Descriptive statistics were calculated for a detailed sample description. Age was categorized by decades. Reliability was assessed by Cronbach’s alpha. Construct validity was determined by Pearson correlations between perceived stress and depression, anxiety, fatigue, procrastination, respectively quality of life. Basing on previous findings suggesting a two-factor solution of the PSS-10, a confirmatory factor analysis (CFA) was calculated. The positively worded items determined the first factor, the negatively worded items the second factor. The model was estimated with the maximum likelihood method approach. Model fit was evaluated by using following model fit indices [34]: Chi-square statistic; the comparative-fit-index (CFI) and the Tucker-Lewis Index (TLI) to describe incremental fit; the root mean square error of approximation (RMSEA) was used as an absolute measure of fit. Values of TLI and CFI close to .95 or higher indicate a better fit. RMSEA should be 0.08 or smaller. Sex differences and interaction effects with demographic variables were tested by means of a two-factorial ANOVA with Scheffé test (total PSS-10 score as the dependent variable; sex and one demographic variable as independent variables). Effect sizes were calculated for *t*-tests (Cohen’s *d*) and for ANOVA (η^2^). According to Cohen [35] effect sizes were considered as small (*d* ≥ .2; η^2^ = .01), moderate (*d* ≥ .5; η^2^ = .06) or large (*d* ≥ .8; η^2^ = .14). We performed a forward stepwise linear regression defining the sum score of the PSS-10 as outcome variable. We included sex, age, anxiety, depression, procrastination, fatigue and life satisfaction as predictor variables. In order to provide differentiated norm values, mean sum-scores, standard deviations, and percentiles of each factor of the PSS-10 were analyzed separately for sex and age. Because of the large sample size, *p*-values should be interpreted with caution and in connection with effect estimates. We performed calculations by SPSS Version 21.0 and AMOS© 21.0.

## Results

### Reliability and validity

In the present study scores on the Perceived Stress Scale demonstrated good internal consistency (Cronbach alpha = .84). In line with previous research, perceived stress was strongly positively correlated with depression and anxiety, fatigue, as well as procrastination, whereas the correlation between perceived stress and life satisfaction was negative (see Table 2).

**Table 2:** Correlations between PSS-10 and depression, anxiety, fatigue, procrastination and life satisfaction (*N* = 2463)

Scale		*r*
Depression	PHQ-2	.59**
Anxiety	GAD-2	.59**
Fatigue	CBI	.57**
Procrastination	GPS	.42**
Life satisfaction	FLZ S	-.47**

Results are significant at **p < .01

### Factor analyzes and scale item characteristics

Confirmatory factor analysis revealed that both incremental fit indexes (CFI = .96; TLI = .95); and absolute measures of fit indexes were good (RMSEA = .07) for the two-dimensional model (*χ*2 (32, *N* = 2463) = 417.8, *p* < .001). This model contained two related latent factors (r = .47). Standardized factor loadings of factor 1 ranged from .54 to .82; those of factor 2 varied between .74 and .82. The descriptive characteristics and corrected item-total correlation of each item are given in Table 3.

**Table 3:** Item Descriptive Statistics and corrected item-total for the PSS-10

Item	In the last month. how often…	*M*	*SD*	*rIT*
1	…have you been upset because of something that happened unexpectedly?	1.19	.93	.45
2	…have you felt that you were unable to control the important things in your life?	.89	.94	.64
3	…have you felt nervous and “stressed”?	1.41	.99	.46
4	…have you felt confident about your ability to handle your personal problems?	1.38	1.10	.46
5	…have you felt that things were going your way?	1.52	1.01	.58
6	…have you found that you could not cope with all the things that you had to do?	1.01	.92	.62
7	…have you been able to control irritations in your life?	1.40	1.06	.47
8	…you felt that you were on top of things?	1.15	0.96	.60
9	…you been angered because of things that were outside your control?	1.59	1.01	.43
10	…have you felt difficulties were piling up so high that you could not overcome them?	1.00	.98	.69
TS		12.57	6.42	-

*rIT* corrected item correlation, *TS* Total Score

### Socio- demographic characteristics

Descriptive characteristics of general perceived stress in the German population, according to sex and socio-demographic variables, are presented in Table 4. Women scored significantly higher on the PSS-10 than men [*t*(2461) = 4.3, *p* < .001; *d* = .18]. A two-way ANOVA revealed a significant main effect for age (*F[*2,2463] = 5.8). Post hoc analysis using Scheffé test showed that the youngest age group (14-19 years.) and the age group from 40 to 59 years showed a significantly higher level of stress than the oldest age group (≥60 year). There was a main effect on marital status (*F[*4, 2460] = 7.8): Unmarried and divorced participants felt more stressed than married persons. Religion showed a significant effect on perceived stress (*F[*3,2453] = 4.3). Post-hoc analyses indicated that Christians and people without religious denomination scored lower on the PSS-10 than Muslims, even if migration background was considered as covariate in the analysis (*F*[1,2453] = .00; *p* = .94). With regard to education (*F[*4,2395] = 7.5), participants without any qualification felt most stressed. A main effect was found for employment status (*F*[4,2452] = 10.6). Post hoc analysis revealed that unemployed participants reported the highest perceived stress level in comparison to every other employments status apart from participants in training. Further results indicated differences in perceived stress depending on income (*F*[4,2243] = 9.9) in interaction with sex (*F*[4,2243] = 3.1; *p* < 01; η^2^ = .01): Perceived stress increased with lower income. Men with lower income reported more stress than women in the same income level. However, men earning more money felt less stressed than women with comparably high income. As age correlated with income, age was considered as a covariate in the analysis with a significant result (*F*[1,2243] = 7.7; *p* ≤ .01; η^2^ = .004). The main effect for income, however, remained significant (see Table 4). There were no differences regarding children and immigrant background. Besides from the interaction between sex and income, no further interaction with sex was found.

**Table 4:** Perceived stress in the general population according to demographic characteristics

	Total sample (*N* = 2463)		Women (*n* = 1315)		Men (*n* = 1148)		η^2^/sign.^a^
	*M*	*SD*	*M*	*SD*	*M*	*SD*	
Age							.01*
(A) 14-19 yrs.	14.05	6.54	15.02	5.93	13.42	6.88	
(B) 20-39 yrs.	12.74	6.67	13.34	6.75	12.01	6.51	
(C) 40-59 yrs.	12.82	6.42	12.99	6.48	12.61	6.35	
(D) ≥ 60 yrs.	11.94	6.14	12.82	6.08	10.92	6.06	
Marital status							.01*
(A) unmarried	13.41	6.70	14.40	6.64	12.58	6.65	
(B) married	12.07	5.90	12.57	5.77	11.60	6.02	
(C) divorced	13.09	6.84	13.33	6.90	12.77	6.78	
(D) widowed	12.93	6.36	12.86	6.35	13.18	6.45	
Children							
yes	12.88	6.21	13.07	6.08	12.62	6.39	
no	12.48	6.49	13.11	6.54	11.80	3.37	
Migration							
yes	13.62	6.50	13.92	6.43	13.27	6.64	
no	12.54	6.42	13.07	6.42	11.93	6.37	
Religion							.01*
(A) Christian	12.51	6.54	13.09	6.52	11.79	6.05	
(B) Muslim	15.40	6.23	16.00	5.74	14.97	6.61	
(C) other	12.51	5.79	12.97	6.22	11.42	4.62	
(D) no religion	12.48	6.11	12.89	6.09	12.09	6.10	
Education							.01*
(A) <10th grade	12.72	6.48	13.17	6.44	12.24	6.50	
(B) completed 10th grade	12.50	6.37	12.98	6.43	11.82	6.22	
(C) high school	12.60	6.37	13.16	6.68	12.01	6.00	
(D) college/university	11.16	5.79	11.97	5.61	10.44	5.87	
(E) without qualification	15.68	7.22	16.02	6.60	15.26	8.00	
Employment							.02*
(A) Employed	12.32	6.30	12.79	6.34	11.83	6.22	
(B) Student/training	13.72	6.52	15.13	6.37	12.58	6.45	
(C) Unemployed	15.39	7.42	15.05	7.54	15.73	7.32	
(D) Retired	12.14	6.28	13.01	6.28	11.13	6.13	
(E) Household	12.59	6.26	12.53	6.27	13.18	6.40	
Income^b^							.02*
(A) ≤ 750	13.83	6.54	13.47	6.33	15.04	7.11	
(B) 751-1250	13.33	6.64	13.60	6.65	12.86	6.60	
(C) 1251-2000	11.68	6.21	12.11	6.25	11.31	6.16	
(D) 2001-3500	11.77	6.14	13.26	5.84	11.26	6.16	
(E) > 3500	11.36	5.64	13.79	6.94	10.59	5.01	

* = *p* < .01; η^2^ = effect size; ^a^ANOVA main effects; significant Scheffé-tests: age: (A) > (D), (C) > (D); marital status: (A), (C) > (B); religion: (B) > (A), (D); education: (E) > (A), (B), (C), (D); (A) > (D); employment: (C) > (A), (D), (E);; income: (A) > (C), (D); (B) > (C), (D); ^b^€/month per person

### Predictors of perceived stress

As Table 5 shows, younger age and life satisfaction, as well as higher scores on anxiety, fatigue and procrastination predicted perceived stress in a multivariate analysis, explaining 50 % of variance.

**Table 5:** Predictors of PSS-10 (*N* = 2463)

	β	*t*	Sign.
Age	-.08	-5.2	***
Anxiety (GAD-2)	.29	16.0	***
Life satisfaction (FLZ-M)	-.16	-9.1	***
Procrastination (GPS-K)	.18	11.0	***
Fatigue (CBI)	.28	15.2	***

β *=* regression coefficients*; R* ^*2*^ = .50; *** *p* ≤ .001

### Norm values

Basing on the results of the CFA, Tables 6 and 7 provide mean sum-scores, standard deviations, and percentiles of both factors of the PSS-10, separately for sex and age categorized by decades.

**Table 6:** Means, standard deviations and percentiles of the PSS-10 factor “perceived helplessness” according to sex and age

	Total (*N* = 2463)						
age group	14-19	20-29	30-39	40-49	50-59	60-69	70+
Mean	8.0	7.6	7.1	7.5	7.2	6.1	6.9
SD	4.8	4.7	4.5	4.3	4.4	3.9	4.1
5 %	1	1	1	1	1	1	1
25 %	4	4	4	4	4	3	4
50 %	8	7	7	7	7	6	6
75 %	11	11	10	10	10	9	9
95 %	16	16	16	16	15	13	15
	Female (*n* = 1315)						
age group	14-19	20-29	30-39	40-49	50-59	60-69	70+
Mean	8.7	8.2	7.4	7.6	7.5	6.9	7.4
SD	4.2	5.0	4.6	4.2	4.7	3.7	4.2
5 %	1	1	1	1	1	1	1
25 %	6	4	4	5	4	5	4
50 %	9	8	7	7	7	7	7
75 %	12	12	10	10	11	9	10
95 %	15	17	16	15	15	13	15
	Male (*n* = 1148)						
age group	14-19	20-29	30-39	40-49	50-59	60-69	70+
Mean	7.5	6.9	6.8	7.5	6.8	5.3	6.2
SD	5.1	4.3	4.4	4.4	4.1	3.8	3.9
5 %	1	1	1	1	1	0	1
25 %	4	4	3	4	3	2	3
50 %	6	6	7	7	7	5	6
75 %	11	10	9	10	10	8	9
95 %	17	15	16	16	14	12	15

**Table 7:** Means, standard deviations and percentiles of the PSS-10 factor “perceived self-efficacy” according to sex and age

	Total (*N* = 2463)						
age group	14-19	20-29	30-39	40-49	50-59	60-69	70+
Mean	6.1	5.8	5.0	5.5	5.4	5.1	5.9
SD	3.2	3.7	3.4	3.4	3.5	3.4	3.3
5 %	1	0	0	1	0	0	1
25 %	4	3	3	3	3	3	4
50 %	6	5	5	5	5	4	6
75 %	8	8	7	8	8	7	8
95 %	11	13	12	12	12	12	12
	Female (*n* = 1315)						
age group	14-19	20-29	30-39	40-49	50-59	60-69	70+
Mean	6.3	6.2	5.1	5.4	5.4	5.2	6.1
SD	3.0	3.6	3.3	3.2	3.4	3.3	3.2
5 %	2	1	0	1	0	0	1
25 %	4	4	3	3	3	3	4
50 %	7	6	5	5	5	5	6
75 %	8	8	7	7	8	7	8
95 %	11	13	11	11	11	12	12
	Male (*n* = 1148)						
age group	14-19	20-29	30-39	40-49	50-59	60-69	70+
Mean	5.9	5.4	5.0	5.7	5.3	5.0	5.6
SD	3.3	3.8	3.5	3.7	3.7	3.5	3.6
5 %	0	0	0	0	0	1	0
25 %	4	3	3	3	3	3	3
50 %	6	5	4	5	5	4	5
75 %	8	7	7	8	8	7	8
95 %	11	13	12	12	12	12	13

## Discussion

This is the first study evaluating psychometrically the established PSS-10 in a representative German community sample covering a broad spectrum of demographic characteristics.

The results of the current study indicated good internal consistency and construct validity of the PSS-10. Perceived stress correlated strongly positively with depression, anxiety, fatigue, and procrastination and negatively with life satisfaction. Consistent with previous research [10, 13], the findings support the construct validity of the PSS. The suggested bi-dimensional model of the PSS-10 was confirmed by CFA consistent with previous studies (e.g. [36]). While Cohen and Williams [11] considered the distinction between the two factors as irrelevant and defined perceived stress as an unidimensional construct, Hewitt and colleagues [21] suggested that the scale covered two separate factors, measuring general stress and the ability to cope. Thus, Roberti and colleagues [37] labelled the first factor “perceived helplessness” and the second factor “perceived self-efficacy”. In line with the concept of primary and secondary appraisal within the process of coping with stressful events [9], the first factor emphasizes the individual’s reaction to stress, while the second factor refers to the self-assessed ability to cope with these stressors. In line with the multidimensional conceptualization of perceived stress, norm values are presented separately for both factors.

The level of general perceived stress differed between demographic groups. As psychological stress is associated with various diseases, the identification of highly stressed subpopulations is essential. As hypothesized, women reported more perceived stress than men. Perceived stress was highest among younger, unmarried and divorced, unemployed and less educated participants.

The reported higher level of stress among females is consistent with results of other studies in diverse countries [4, 20–22, 36, 38], although Cohen and collogues found no differences in their original study from 1983. Possible explanations for this finding have been discussed controversially taking social, biological and psychological hypotheses into account (e.g. [23, 39]). We found that women with higher income reported more stress than men with comparable salaries. Working women do not seem to feel more stressed at work per se, but are more likely exposed to non-work stressors compared to males [40]. Hence, from a social-psychological perspective, defined gender roles and expected demands in society may have an impact on perceived stress. However, the experience of stress seems to differ between sexes: women tend to feel emotionally exhausted, whereas men feel more depersonalized [24]. As the PSS includes no item assessing psychological stress as depersonalization, the level of stress among men might be underestimated in the current study. Therefore, further research is required to improve the understanding of the association between sex and perceived stress and its causes by measuring different ways of stress experiences.

In regard to age, in the current study younger participants felt significantly more stressed than their older counterparts in agreement with Cohen and Janicki-Devert’s [25] findings assessing the PSS in a national survey. A possible explanation for this result is provided by Carstensen's Socioemotional Selectivity Theory [41, 42]: Aging people prioritize to satisfy their social and emotional goals by focusing more on positive and less on negative emotions (e.g. reduced exposure to daily hassles [43]). The finding, that married persons feel less stressed, is consistent with evidence that social support reduced psychological stress (e.g. [44]). Regarding religion Muslim participants showed higher scores on the PSS-10 than Christians and participants unaffiliated with any religion. As a minority group, Muslims might be more exposed to social and psychological stressors including discrimination (e.g. [45, 46]) suggesting that rather the minority status than the denomination itself is linked to higher stress. In accordance with previous findings [25], and our hypotheses, the present study has demonstrated that perceived stress increased with lower education, income and employment status. This result may be explained by findings that unemployment, which is associated with lower income and lower education [47], may lead to declined psychological and physical well-being, and increased exposure to stressful events ([48]; see for a meta-analysis: [49]). In this context coping strategies and coping resources play a crucial role whose development and adaptive use can be impaired by socioeconomic status [50, 51]. Interestingly, the results of the present study suggested that people in training and students reported a slightly higher level of stress. Considering Lee’s [13] objection that the PSS has been mainly explored in students’ samples, this result underlines the importance to validate the scale in a representative sample in order to avoid misinterpretations. Moreover, in contrast to a former study [38], participants with children did not feel more stressed than their childless counterparts. This inconsistency may be due to the different samples as Lesage and colleagues assessed the PSS only in a working population. No differences were found regarding migration background. Interestingly, multiple regression revealed procrastination as a significant predictor for perceived stress giving further evidence for the association between procrastination and perceived stress [26, 52].

Finally, important limitations need to be considered. Data are limited to self-report which can lead to a response bias. Although significant differences were found, effect sizes were small. The effect of religious denomination is to be interpreted with caution due to a low number of Muslims in the sample. Because of the cross sectional study design the results do not allow drawing any causal conclusion. Therefore future longitudinal studies are needed to detect developments and changes of perceived stress over the time. However, to the best of our knowledge, the present study provided norm values for the PSS-10 basing on a large and randomly selected population, which so far only exists for the American [4, 11] and Swedish population [20].

## Conclusion

In summary, the PSS-10 is a reliable and valid instrument for assessing perceived stress. The scale is easy to complete and can be administered in only a few minutes providing an economic measure for research and practice. It is important, however, to bear in mind that some subpopulations are more likely to experience stress as the present study showed. The dependency of the perceived stress level on socio-demographic variables, particularly age and sex, implies to use differentiated norm values, which are provided in this paper.

## Abbreviations

ESOMAR: European Society for Opinion and Market Research
GCP: good clinical practice
ICC: International Chamber of Commerce
ICH: International Conference on Harmonisation of Technical Requirements for Registration of Pharmaceuticals for Human Use
